# Complete Vision Loss following Orbital Cellulitis Secondary to Acute Dacryocystitis

**DOI:** 10.1155/2016/9630698

**Published:** 2016-10-10

**Authors:** Margaret L. Pfeiffer, Alexander Hacopian, Helen Merritt, Margaret E. Phillips, Karina Richani

**Affiliations:** ^1^Ruiz Department of Ophthalmology and Visual Science, The University of Texas Health Science Center at Houston, Houston, TX, USA; ^2^Robert Cizik Eye Clinic, Houston, TX, USA; ^3^Medical School, The University of Texas Health Science Center at Houston, Houston, TX, USA

## Abstract

We present a case of a 50-year-old woman with acute dacryocystitis that was complicated by posterior rupture of the lacrimal sac causing an orbital cellulitis with subsequent visual acuity of no light perception. Upon presentation, she was immediately started on broad-spectrum antibiotics and underwent surgical incision and drainage of the lacrimal sac abscess but never regained vision. There are 4 cases in the literature of permanent severe vision loss from acute dacryocystitis. Prompt diagnosis and close monitoring of acute dacryocystitis are therefore essential to prevent extension into the orbit and possible optic nerve compromise.

## 1. Introduction

Dacryocystitis is an infection of the lacrimal sac secondary to obstruction of the nasolacrimal duct. Acute dacryocystitis typically causes preseptal cellulitis but is rarely associated with orbital cellulitis. Typically orbital cellulitis responds to systemic antibiotic therapy and surgical drainage without permanent optic nerve compromise.

## 2. Case Description

**Figure 1:**
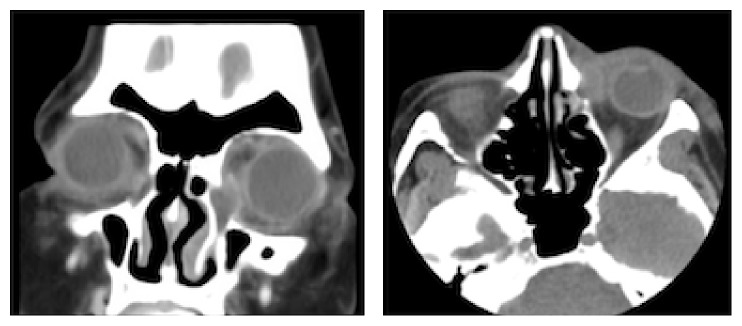
Coronal and axial CT of the orbits with contrast showing an enlarged left lacrimal sac and preseptal inflammatory changes consistent with acute dacryocystitis with preseptal cellulitis.

A 50-year-old Hispanic woman with no significant past medical history presented to our emergency department with a 1-week history of left eye pain and 1-day history of decreased vision in her left eye. She had visited another hospital 2 days prior to presentation, where she was diagnosed with acute dacryocystitis and preseptal cellulitis, based on her clinical exam and orbital imaging (Figure 1). She was discharged home on oral amoxicillin/clavulanic acid and asked to follow up with ophthalmology as an outpatient. Despite compliance with the prescribed oral antibiotics, her symptoms worsened significantly, thus prompting presentation to our institution.

**Figure 2:**
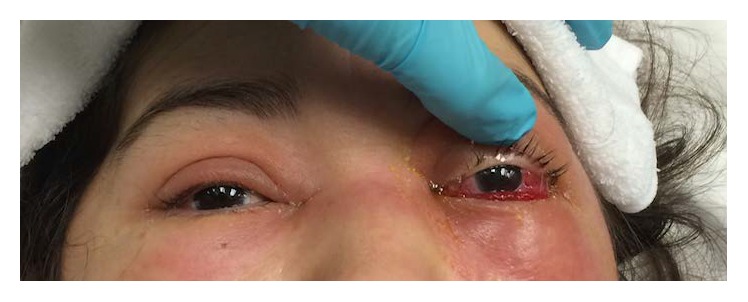
External photograph showing left upper and lower eyelid edema and erythema and conjunctival chemosis.

On evaluation, visual acuity was 20/25 OD and no light perception OS. The left pupil was 4 mm and nonreactive with a left relative afferent pupillary defect. She had left proptosis with complete ophthalmoplegia. She also had left upper and lower eyelid edema and erythema and significant resistance to retropulsion of the left globe (Figure 2). Intraocular pressure was 80 mm Hg OS.

**Figure 3:**
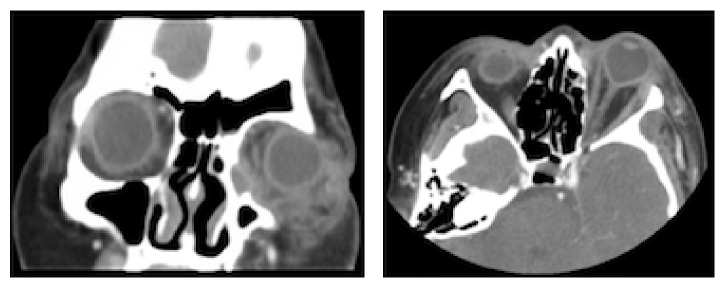
Coronal and axial CT of the orbits with contrast showing an enlarged left lacrimal sac, a large complex fluid collection in the inferomedial left orbit, and severe tenting of the posterior globe. The lacrimal sac is smaller in size compared to Figure 1 suggestive of posterior rupture into the orbit causing decompression of the sac.

Computed tomography (CT) of the orbits with contrast showed an enlarged left lacrimal sac, a large complex fluid collection in the inferomedial left orbit, and severe tenting of the posterior globe (Figure 3). There was an interval decrease in the size of the left lacrimal sac compared to prior CT, suggestive of posterior rupture into the orbit with subsequent decompression of the sac.

The patient was started promptly on intravenous vancomycin and piperacillin-tazobactam and taken emergently to the operating room. Significant purulent material was drained from the lacrimal sac, which was noted to have ruptured posteriorly into the orbit. After drainage of the lacrimal sac and medial aspect of the orbit, no additional purulence was noted. A lateral canthotomy and inferior cantholysis was performed.

Postoperatively, the patient had significant reduction in proptosis, resistance to retropulsion, eyelid edema, and erythema. As there was no improvement in motility after admission day 4, intravenous methylprednisolone was administered with subsequent significant improvement in motility. Unfortunately, her visual acuity remained at no light perception.

Intraoperative cultures were positive for* Staphylococcus epidermidis*. She was discharged on postoperative day 6 with a peripherally inserted central catheter and a 2-week course of intravenous ceftaroline and oral metronidazole. Three months after discharge, she underwent uncomplicated left external dacryocystorhinostomy and lateral canthoplasty. Crawford tubes were removed 12 weeks postoperatively. At 6 months of follow-up, she was without tearing.

## 3. Discussion

Acute dacryocystitis can commonly cause preseptal cellulitis but is rarely associated with orbital cellulitis. In such cases, associated orbital cellulitis typically does not cause optic nerve compromise, and patients do well after systemic antibiotics and surgical treatment [1–5].

There are only 4 cases of acute dacryocystitis leading to severe, permanent vision loss reported in the literature [1, 6, 7]. In one patient, no light perception vision was attributed to central retinal artery occlusion [6]. A second case was a 38-year-old woman with recurrent dacryocystitis who presented with no light perception and a medial subperiosteal abscess secondary to* Staphylococcus aureus* [7]. She underwent emergent orbitotomy and incision and drainage of the subperiosteal abscess and dacryocystorhinostomy. The third case was a 64-year-old woman with a history of alcoholism who, while being hospitalized for hepatic failure, developed acute deterioration in visual acuity to light perception with a large posteromedial orbital abscess [7]. She was treated with intravenous vancomycin, but surgical intervention was postponed due to her acute medical issues. She eventually underwent orbitotomy with incision and drainage of the orbital abscess and dacryocystorhinostomy. Cultures were positive for nonenterococcal group D streptococci,* Streptococcus viridans*, and* Streptococcus intermedius*. The fourth case was a 65-year-old woman with a history of recurrent dacryocystitis and nasolacrimal duct obstruction who presented with visual acuity of no light perception and a posteromedial orbital abscess [1]. She underwent orbitotomy with incision and drainage of the abscess.

A prior episode of dacryocystitis is accepted as risk factor for orbital spread of the infection. When examining previously reported cases, Kikkawa et al. found an incidence of prior dacryocystitis in 40% of patients presenting with an acute episode and posterior extension [7]. Although our patient had no history of dacryocystitis, it is possible that chronic distention of the lacrimal sac led to weakness and predisposition to posterior rupture.

Kikkawa et al. emphasize the strong anatomical barriers to posterior infectious spread from the lacrimal sac. Although the deep heads of the preseptal and pretarsal orbicularis muscles function as the primary barrier, the insertions of the orbital septum and medial canthal ligaments at the lacrimal crest provide an additional impediment to posterior extension. They note, however, that a breach of these structures provides direct access into the intraconal space, making abscess formation immediately sight-threatening and requiring urgent intervention [7].

Our case underscores the need for prompt diagnosis and close monitoring of acute dacryocystitis. If an abscess is present, we recommend incision and drainage in an attempt to prevent rupture of the lacrimal sac and posterior extension into the orbit, with dacryocystorhinostomy providing the definitive treatment. If orbital involvement does develop, optic nerve compromise may progress quickly and be irreversible. As seen in our case, the patient had no light perception from severe orbital infection with acute dacryocystitis. Despite aggressive treatment at the time of presentation to our institution, she did not recover vision in the affected eye.
